# 1-Benzyl­piperidin-4-one *O*-(2-bromo­benz­yl)oxime

**DOI:** 10.1107/S1600536812040263

**Published:** 2012-09-29

**Authors:** Rodolfo Moreno-Fuquen, Alix E. Loaiza, John Diaz-Velandia, Alan R. Kennedy, Catriona A. Morrison

**Affiliations:** a Departamento de Química, Facultad de Ciencias, Universidad del Valle, Apartado 25360, Santiago de Cali, Colombia; b Laboratorio de Sintesis Orgánica, Facultad de Ciencias, Pontificia Universidad Javeriana, Bogota, DC, Colombia; c WestCHEM, Department of Pure and Applied Chemistry, University of Strathclyde, 295 Cathedral Street, Glasgow G1 1XL, Scotland

## Abstract

In the title mol­ecule, C_19_H_21_BrN_2_O, the piperidone ring adopts a chair conformation with a total puckering amplitude *Q*_T_ of 0.554 (2) Å. The dihedral angle between the benzene rings is 64.10 (7)°. There are no significant inter­molecular inter­actions.

## Related literature

For the use of the oxime function in organic synthesis, see: Mikhaleva *et al.* (2006 ▶). For properties of the oxime function, see: Parthiban *et al.* (2011 ▶); Jayabharathi *et al.* (2011 ▶); Picard *et al.* (2000 ▶); For related structures, see: Parthiban *et al.* (2009 ▶); For details of ring-puckering conformational analysis, see: Cremer & Pople (1975 ▶).
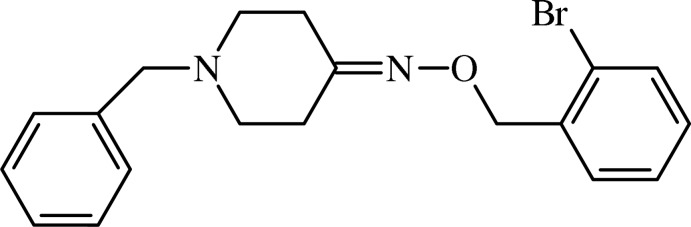


## Experimental

### 

#### Crystal data

C_19_H_21_BrN_2_O
*M* *_r_* = 373.29
Monoclinic, 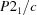

*a* = 21.1586 (5) Å
*b* = 5.6731 (2) Å
*c* = 14.6425 (4) Å
β = 103.037 (3)°
*V* = 1712.31 (9) Å^3^
*Z* = 4
Mo *K*α radiation
μ = 2.41 mm^−1^
*T* = 123 K
0.40 × 0.12 × 0.05 mm

#### Data collection

Oxford Diffraction Xcalibur E diffractometer
Absorption correction: multi-scan (*CrysAlis PRO*; Oxford Diffraction, 2010 ▶) *T*_min_ = 0.448, *T*_max_ = 1.000
9090 measured reflections
4525 independent reflections
3556 reflections with *I* > 2σ(*I*)
*R*_int_ = 0.032

#### Refinement

*R*[*F* ^2^ > 2σ(*F* ^2^)] = 0.037
*wR*(*F* ^2^) = 0.078
*S* = 1.03
4525 reflections
208 parameters
H-atom parameters constrained
Δρ_max_ = 0.45 e Å^−3^
Δρ_min_ = −0.69 e Å^−3^

### 

Data collection: *CrysAlis PRO* (Oxford Diffraction, 2010 ▶); cell refinement: *CrysAlis PRO*; data reduction: *CrysAlis PRO*; program(s) used to solve structure: *SIR92* (Burla *et al.*, 2005 ▶); program(s) used to refine structure: *SHELXL97* (Sheldrick, 2008 ▶); molecular graphics: *ORTEP-3 for Windows* (Farrugia, 1997 ▶); software used to prepare material for publication: *WinGX* (Farrugia, 1999 ▶).

## Supplementary Material

Crystal structure: contains datablock(s) I, global. DOI: 10.1107/S1600536812040263/gg2101sup1.cif

Structure factors: contains datablock(s) I. DOI: 10.1107/S1600536812040263/gg2101Isup2.hkl

Supplementary material file. DOI: 10.1107/S1600536812040263/gg2101Isup3.cml

Additional supplementary materials:  crystallographic information; 3D view; checkCIF report

## Figures and Tables

**Fig. 1.**
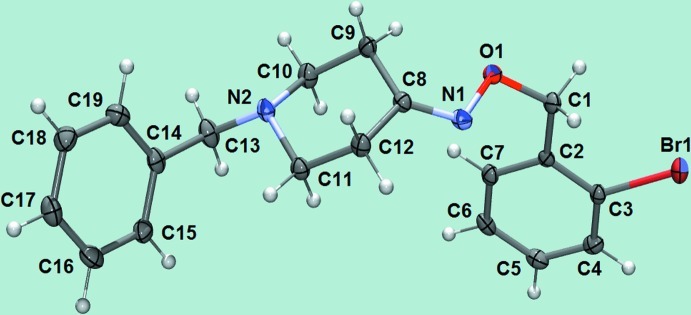
An ORTEP-3 (Farrugia, 1997) plot of (I) with displacement ellipsoids drawn at the 50% probability level. H atoms are shown as spheres of arbitrary radius.

## supplementary crystallographic information

### Comment

The oxime function is a versatile intermediate in organic synthesis, for example, it can be readily transformed into important groups such as carbonyl, amino, nitro, cyano and can be used as a convenient protective group (Mikhaleva *et al.*, 2006). The oxime function is also an important pharmacophore group, particularly oximes of the piperidone and their ethers possess a wide spectrum of biological activity such as cytotoxic (Parthiban *et al.*, 2011), antimicrobial (Jayabharathi *et al.*, 2011) and as an inhibitor of steroid-5-reductase (Picard *et al.*, 2000). In our research group, we are interested in the synthesis of nitrogen containing compounds with potential biological activity such as oximes and isoxazoles. In order to accomplish this objective the 1-benzylpiperidin-4-one *O*-2-bromobenzyl oxime (I) was synthesized with the aim of evaluating its *in vitro* antimicrobial activity. The molecular structure of I is shown in Fig. 1. A single crystal XRD study has been carried out for (I) to confirm the sterochemistry stablished by NMR studies. Analisis of torsion angles, and least-square plane calculation, indicate that piperidone ring adopts a chair conformation with the smallest displacement parameters q_2_= 0.078 (2) and q_3_= 0.549 (2) Å, total puckering amplitude, Q_T_= 0.554 (2) Å and φ_2_= -176 (2)° (Cremer & Pople, 1975). This conformational behavior is similar to that reported by other similar systems (Parthiban *et al.*, 2009). The least-squares fit of two phenyl rings C2/C3/C4/C5/C6/C7 and C14/C15/C16/C17/C18/C19 with a r.m.s deviation of fitted atoms of 0.0.009 and 0.004 Å respectively, shows a dihedral angle of 64.10 (7)° between the rings. The crystal packing shows no classical hydrogen bonds.

### Experimental

In a two necks round bottom flask a mixture of 1-benzylpiperidin-4-one oxime (204 mg, 1 mmol), NaOH (80 mg, 2 mmol) in dry acetone (1.5 ml) was refluxed for 30 minutes, and then 2-bromobenzyl bromide (275 mg, 1,1 mmol) was added and subsequently stirred for 3 h. The reaction mixture was neutralized with acetic acid, extracted with AcOEt and dried with anhydrous Na_2_SO_4_. The combined organic layers were evaporated under low pressure to dryness. Purification of the crude mixture by flash column chromatography with 15% (*v*/*v*) AcOEt/hexane yielded compound 1 as a pale yellow solid (250 mg, 67% yield) mp 340.5 - 342.0 K. 1-benzylpiperidin-4-one*O*-2-bromobenzyl oxime ^1^H NMR (300 MHz) δ, 7.56 (dd, 1H, Ar—H), 7.43–7.27 (m, 7H, Ar—H), 7.17 (td, 1H, Ar—H), 5.16 (s, 2H, O—CH_2_—Ar) 3.58 (s, 2H, N—CH_2_—Ph), 2.73 (t, 2H, N=C—CH_2_), 2.59 (t, 2H, N—CH_2_), 2.56 (t, 2H, N—CH_2_), 2.39 (t, 2H, N=C—CH_2_). ^13^C-NMR δ, 158.3 (C=N), 138.0 (C), 137.5(C), 132.5 (Ar—H), 129.2, 129.1, 128.9, 128.3, 127.2, 122.7 (C—Br), 74.5 (O—CH_2_—Ar), 62.5 (N—CH_2_—Ar), 53.5 (N—CH_2_), 52.4 (N—CH_2_), 31.4 (N=C—CH_2_), 25.5 (N=C—CH_2_). MS—EI *M*^+^ m/*z*: 372.1, 374.1, 100%: 91.1.

### Refinement

The H-atoms were positioned geometrically [C—H= 0.95 Å for aromatic and C—H = 0.99 Å for methylene, and with *U*_iso_(H) (1.2 and 1.5 x *U*_eq_ of the parent atom respectively].

### Crystal data

**Table:**

C_19_H_21_BrN_2_O	*F*(000) = 768
*M**_r_* = 373.29	*D*_x_ = 1.448 Mg m^−^^3^
Monoclinic, *P*2_1_/*c*	Melting point: 341(1) K
Hall symbol: -P 2ybc	Mo *K*α radiation, λ = 0.71073 Å
*a* = 21.1586 (5) Å	Cell parameters from 4022 reflections
*b* = 5.6731 (2) Å	θ = 3.2–30.0°
*c* = 14.6425 (4) Å	µ = 2.41 mm^−^^1^
β = 103.037 (3)°	*T* = 123 K
*V* = 1712.31 (9) Å^3^	Cut from large needle, colourless
*Z* = 4	0.40 × 0.12 × 0.05 mm

### Data collection

**Table:**

Oxford Diffraction Xcalibur E diffractometer	4525 independent reflections
Radiation source: fine-focus sealed tube	3556 reflections with *I* > 2σ(*I*)
Graphite monochromator	*R*_int_ = 0.032
ω scans	θ_max_ = 30.0°, θ_min_ = 3.2°
Absorption correction: multi-scan (*CrysAlis PRO*; Oxford Diffraction, 2010)	*h* = −29→28
*T*_min_ = 0.448, *T*_max_ = 1.000	*k* = −7→7
9090 measured reflections	*l* = −20→18

### Refinement

**Table:**

Refinement on *F*^2^	Primary atom site location: structure-invariant direct methods
Least-squares matrix: full	Secondary atom site location: difference Fourier map
*R*[*F*^2^ > 2σ(*F*^2^)] = 0.037	Hydrogen site location: inferred from neighbouring sites
*wR*(*F*^2^) = 0.078	H-atom parameters constrained
*S* = 1.03	*w* = 1/[σ^2^(*F*_o_^2^) + (0.0294*P*)^2^ + 0.3261*P*] where *P* = (*F*_o_^2^ + 2*F*_c_^2^)/3
4525 reflections	(Δ/σ)_max_ < 0.001
208 parameters	Δρ_max_ = 0.45 e Å^−^^3^
0 restraints	Δρ_min_ = −0.69 e Å^−^^3^

### Special details

**Table:**

Experimental. Absorption correction: CrysAlisPro, Oxford Diffraction Ltd., Version 1.171.34.40 (release 27-08-2010 CrysAlis171 .NET) (compiled Aug 27 2010,11:50:40) Empirical absorption correction using spherical harmonics, implemented in SCALE3 ABSPACK scaling algorithm.
Geometry. All e.s.d.'s (except the e.s.d. in the dihedral angle between two l.s. planes) are estimated using the full covariance matrix. The cell e.s.d.'s are taken into account individually in the estimation of e.s.d.'s in distances, angles and torsion angles; correlations between e.s.d.'s in cell parameters are only used when they are defined by crystal symmetry. An approximate (isotropic) treatment of cell e.s.d.'s is used for estimating e.s.d.'s involving l.s. planes.
Refinement. Refinement of *F*^2^ against ALL reflections. The weighted *R*-factor *wR* and goodness of fit *S* are based on *F*^2^, conventional *R*-factors *R* are based on *F*, with *F* set to zero for negative *F*^2^. The threshold expression of *F*^2^ > σ(*F*^2^) is used only for calculating *R*-factors(gt) *etc*. and is not relevant to the choice of reflections for refinement. *R*-factors based on *F*^2^ are statistically about twice as large as those based on *F*, and *R*- factors based on ALL data will be even larger.

### Fractional atomic coordinates and isotropic or equivalent isotropic displacement parameters (Å^2^)

**Table:**

	*x*	*y*	*z*	*U*_iso_*/*U*_eq_	
Br1	0.089592 (10)	0.38045 (4)	0.610086 (15)	0.02227 (8)	
O1	0.19320 (7)	0.9868 (3)	0.78376 (10)	0.0186 (3)	
N1	0.25028 (8)	0.8872 (3)	0.84339 (13)	0.0182 (4)	
N2	0.31211 (8)	1.2442 (3)	1.08828 (12)	0.0178 (4)	
C1	0.15948 (10)	0.8022 (4)	0.72769 (15)	0.0185 (5)	
H1A	0.1912	0.7023	0.7052	0.022*	
H1B	0.1299	0.8716	0.6721	0.022*	
C2	0.12070 (9)	0.6498 (4)	0.77892 (14)	0.0141 (4)	
C3	0.08859 (9)	0.4501 (4)	0.73724 (14)	0.0155 (4)	
C4	0.05440 (10)	0.3014 (4)	0.78332 (16)	0.0200 (5)	
H4	0.0338	0.1645	0.7531	0.024*	
C5	0.05072 (10)	0.3551 (4)	0.87379 (16)	0.0223 (5)	
H5	0.0278	0.2538	0.9067	0.027*	
C6	0.08033 (10)	0.5564 (4)	0.91680 (15)	0.0212 (5)	
H6	0.0769	0.5950	0.9786	0.025*	
C7	0.11498 (10)	0.7015 (4)	0.86974 (15)	0.0183 (5)	
H7	0.1353	0.8389	0.9000	0.022*	
C8	0.27666 (10)	1.0359 (4)	0.90613 (15)	0.0164 (4)	
C9	0.25232 (10)	1.2752 (4)	0.92308 (15)	0.0202 (5)	
H9A	0.2085	1.2990	0.8827	0.024*	
H9B	0.2815	1.3968	0.9068	0.024*	
C10	0.24949 (10)	1.3006 (4)	1.02568 (15)	0.0214 (5)	
H10A	0.2372	1.4643	1.0375	0.026*	
H10B	0.2158	1.1939	1.0393	0.026*	
C11	0.32969 (10)	0.9998 (4)	1.07409 (15)	0.0188 (5)	
H11A	0.2957	0.8934	1.0871	0.023*	
H11B	0.3710	0.9610	1.1187	0.023*	
C12	0.33726 (10)	0.9598 (4)	0.97427 (15)	0.0188 (5)	
H12A	0.3747	1.0512	0.9634	0.023*	
H12B	0.3455	0.7907	0.9649	0.023*	
C13	0.30752 (11)	1.2827 (5)	1.18558 (15)	0.0246 (5)	
H13A	0.2778	1.1639	1.2024	0.030*	
H13B	0.2886	1.4403	1.1907	0.030*	
C14	0.37229 (10)	1.2669 (4)	1.25436 (15)	0.0198 (5)	
C15	0.38856 (11)	1.0734 (4)	1.31350 (15)	0.0222 (5)	
H15	0.3588	0.9467	1.3102	0.027*	
C16	0.44798 (12)	1.0645 (4)	1.37719 (16)	0.0258 (5)	
H16	0.4587	0.9316	1.4171	0.031*	
C17	0.49166 (11)	1.2485 (5)	1.38284 (16)	0.0263 (5)	
H17	0.5321	1.2433	1.4272	0.032*	
C18	0.47616 (11)	1.4401 (4)	1.32351 (17)	0.0249 (5)	
H18	0.5062	1.5658	1.3263	0.030*	
C19	0.41685 (11)	1.4481 (4)	1.26022 (16)	0.0229 (5)	
H19	0.4065	1.5805	1.2199	0.027*	

### Atomic displacement parameters (Å^2^)

**Table:**

	*U*^11^	*U*^22^	*U*^33^	*U*^12^	*U*^13^	*U*^23^
Br1	0.02297 (11)	0.02514 (14)	0.01779 (12)	−0.00154 (10)	0.00266 (8)	−0.00613 (10)
O1	0.0158 (7)	0.0169 (8)	0.0189 (8)	−0.0016 (6)	−0.0045 (6)	0.0019 (7)
N1	0.0136 (8)	0.0198 (10)	0.0194 (9)	0.0013 (7)	−0.0002 (7)	0.0026 (8)
N2	0.0140 (8)	0.0219 (10)	0.0160 (9)	0.0038 (7)	0.0006 (7)	−0.0017 (8)
C1	0.0175 (10)	0.0189 (11)	0.0169 (11)	−0.0036 (9)	−0.0011 (8)	−0.0009 (9)
C2	0.0122 (9)	0.0140 (11)	0.0142 (10)	0.0022 (8)	−0.0013 (7)	0.0010 (9)
C3	0.0129 (9)	0.0176 (11)	0.0143 (10)	0.0025 (8)	−0.0006 (8)	−0.0020 (9)
C4	0.0155 (10)	0.0183 (11)	0.0242 (12)	0.0008 (9)	0.0005 (9)	0.0012 (10)
C5	0.0188 (10)	0.0258 (13)	0.0219 (11)	−0.0007 (9)	0.0033 (9)	0.0071 (10)
C6	0.0207 (11)	0.0283 (13)	0.0139 (10)	0.0031 (9)	0.0025 (8)	0.0033 (10)
C7	0.0159 (10)	0.0199 (11)	0.0167 (11)	0.0003 (9)	−0.0015 (8)	−0.0018 (9)
C8	0.0147 (10)	0.0184 (11)	0.0159 (10)	−0.0007 (8)	0.0029 (8)	0.0007 (9)
C9	0.0203 (10)	0.0186 (12)	0.0183 (11)	0.0012 (9)	−0.0027 (9)	−0.0008 (10)
C10	0.0158 (10)	0.0253 (12)	0.0215 (11)	0.0039 (9)	0.0008 (9)	−0.0029 (10)
C11	0.0160 (10)	0.0214 (12)	0.0175 (11)	0.0010 (9)	0.0006 (8)	0.0001 (10)
C12	0.0139 (10)	0.0209 (12)	0.0199 (11)	0.0030 (9)	0.0004 (8)	−0.0018 (10)
C13	0.0197 (11)	0.0363 (14)	0.0179 (11)	0.0038 (10)	0.0046 (9)	−0.0026 (11)
C14	0.0184 (10)	0.0271 (13)	0.0144 (10)	0.0033 (9)	0.0050 (8)	−0.0037 (10)
C15	0.0264 (11)	0.0232 (13)	0.0176 (11)	−0.0035 (9)	0.0060 (9)	−0.0023 (10)
C16	0.0311 (13)	0.0288 (14)	0.0179 (11)	0.0036 (10)	0.0059 (10)	0.0048 (10)
C17	0.0211 (11)	0.0360 (15)	0.0199 (12)	0.0007 (10)	0.0010 (9)	−0.0027 (12)
C18	0.0242 (12)	0.0246 (13)	0.0258 (13)	−0.0055 (10)	0.0054 (10)	−0.0013 (11)
C19	0.0241 (11)	0.0227 (12)	0.0223 (12)	0.0039 (9)	0.0059 (9)	0.0038 (10)

### Geometric parameters (Å, º)

**Table:**

Br1—C3	1.908 (2)	C9—H9A	0.9900
O1—C1	1.420 (2)	C9—H9B	0.9900
O1—N1	1.437 (2)	C10—H10A	0.9900
N1—C8	1.279 (3)	C10—H10B	0.9900
N2—C11	1.462 (3)	C11—C12	1.523 (3)
N2—C13	1.466 (3)	C11—H11A	0.9900
N2—C10	1.466 (2)	C11—H11B	0.9900
C1—C2	1.504 (3)	C12—H12A	0.9900
C1—H1A	0.9900	C12—H12B	0.9900
C1—H1B	0.9900	C13—C14	1.509 (3)
C2—C3	1.389 (3)	C13—H13A	0.9900
C2—C7	1.393 (3)	C13—H13B	0.9900
C3—C4	1.382 (3)	C14—C19	1.384 (3)
C4—C5	1.379 (3)	C14—C15	1.392 (3)
C4—H4	0.9500	C15—C16	1.387 (3)
C5—C6	1.384 (3)	C15—H15	0.9500
C5—H5	0.9500	C16—C17	1.384 (3)
C6—C7	1.385 (3)	C16—H16	0.9500
C6—H6	0.9500	C17—C18	1.384 (3)
C7—H7	0.9500	C17—H17	0.9500
C8—C9	1.492 (3)	C18—C19	1.382 (3)
C8—C12	1.500 (3)	C18—H18	0.9500
C9—C10	1.524 (3)	C19—H19	0.9500
			
C1—O1—N1	107.75 (15)	C9—C10—H10A	109.3
C8—N1—O1	110.33 (17)	N2—C10—H10B	109.3
C11—N2—C13	110.74 (18)	C9—C10—H10B	109.3
C11—N2—C10	109.94 (17)	H10A—C10—H10B	108.0
C13—N2—C10	109.04 (17)	N2—C11—C12	111.28 (18)
O1—C1—C2	113.28 (18)	N2—C11—H11A	109.4
O1—C1—H1A	108.9	C12—C11—H11A	109.4
C2—C1—H1A	108.9	N2—C11—H11B	109.4
O1—C1—H1B	108.9	C12—C11—H11B	109.4
C2—C1—H1B	108.9	H11A—C11—H11B	108.0
H1A—C1—H1B	107.7	C8—C12—C11	109.66 (18)
C3—C2—C7	116.8 (2)	C8—C12—H12A	109.7
C3—C2—C1	121.16 (19)	C11—C12—H12A	109.7
C7—C2—C1	121.99 (19)	C8—C12—H12B	109.7
C4—C3—C2	122.8 (2)	C11—C12—H12B	109.7
C4—C3—Br1	118.18 (16)	H12A—C12—H12B	108.2
C2—C3—Br1	119.04 (16)	N2—C13—C14	112.97 (18)
C5—C4—C3	118.9 (2)	N2—C13—H13A	109.0
C5—C4—H4	120.5	C14—C13—H13A	109.0
C3—C4—H4	120.5	N2—C13—H13B	109.0
C4—C5—C6	120.1 (2)	C14—C13—H13B	109.0
C4—C5—H5	119.9	H13A—C13—H13B	107.8
C6—C5—H5	119.9	C19—C14—C15	118.5 (2)
C5—C6—C7	120.0 (2)	C19—C14—C13	120.0 (2)
C5—C6—H6	120.0	C15—C14—C13	121.4 (2)
C7—C6—H6	120.0	C16—C15—C14	120.4 (2)
C6—C7—C2	121.3 (2)	C16—C15—H15	119.8
C6—C7—H7	119.4	C14—C15—H15	119.8
C2—C7—H7	119.4	C17—C16—C15	120.3 (2)
N1—C8—C9	127.48 (19)	C17—C16—H16	119.9
N1—C8—C12	117.18 (19)	C15—C16—H16	119.9
C9—C8—C12	115.27 (18)	C18—C17—C16	119.6 (2)
C8—C9—C10	109.98 (18)	C18—C17—H17	120.2
C8—C9—H9A	109.7	C16—C17—H17	120.2
C10—C9—H9A	109.7	C19—C18—C17	119.8 (2)
C8—C9—H9B	109.7	C19—C18—H18	120.1
C10—C9—H9B	109.7	C17—C18—H18	120.1
H9A—C9—H9B	108.2	C18—C19—C14	121.3 (2)
N2—C10—C9	111.39 (18)	C18—C19—H19	119.3
N2—C10—H10A	109.3	C14—C19—H19	119.3
			
C1—O1—N1—C8	−169.11 (18)	C13—N2—C10—C9	177.2 (2)
N1—O1—C1—C2	78.7 (2)	C8—C9—C10—N2	53.8 (3)
O1—C1—C2—C3	−175.08 (17)	C13—N2—C11—C12	−177.74 (17)
O1—C1—C2—C7	5.1 (3)	C10—N2—C11—C12	61.7 (2)
C7—C2—C3—C4	−2.5 (3)	N1—C8—C12—C11	−127.6 (2)
C1—C2—C3—C4	177.67 (19)	C9—C8—C12—C11	49.4 (3)
C7—C2—C3—Br1	175.90 (15)	N2—C11—C12—C8	−54.7 (2)
C1—C2—C3—Br1	−3.9 (3)	C11—N2—C13—C14	68.1 (3)
C2—C3—C4—C5	1.4 (3)	C10—N2—C13—C14	−170.8 (2)
Br1—C3—C4—C5	−177.08 (16)	N2—C13—C14—C19	74.1 (3)
C3—C4—C5—C6	0.7 (3)	N2—C13—C14—C15	−106.4 (3)
C4—C5—C6—C7	−1.4 (3)	C19—C14—C15—C16	0.5 (3)
C5—C6—C7—C2	0.2 (3)	C13—C14—C15—C16	−179.0 (2)
C3—C2—C7—C6	1.7 (3)	C14—C15—C16—C17	0.2 (4)
C1—C2—C7—C6	−178.5 (2)	C15—C16—C17—C18	−1.0 (4)
O1—N1—C8—C9	3.7 (3)	C16—C17—C18—C19	1.0 (4)
O1—N1—C8—C12	−179.76 (17)	C17—C18—C19—C14	−0.3 (4)
N1—C8—C9—C10	127.6 (2)	C15—C14—C19—C18	−0.5 (3)
C12—C8—C9—C10	−49.1 (3)	C13—C14—C19—C18	179.0 (2)
C11—N2—C10—C9	−61.2 (2)		
